# Improving the water solubility of cannabidiol using a peptide carrier

**DOI:** 10.55730/1300-0527.3655

**Published:** 2024-02-16

**Authors:** Melek PARLAK KHALILY

**Affiliations:** Department of Basic Science and Health, Hemp Research Institute, Yozgat Bozok University, Yozgat, Turkiye

**Keywords:** Cannabidiol, self-assembling peptides, solubility, encapsulation, cannabis

## Abstract

Cannabidiol (CBD), nonpsychotropic cannabinoid found in *Cannabis sativa*, is a very promising drug candidate offering many differential effects such as sedative, antiinflammatory, antioxidant, and neuroprotective properties. Nevertheless, the therapeutic use of CBD is hindered by its lack of water solubility and relatively low bioavailability. Various carriers have been used to address the solubility issues of CBD and other highly lipophilic drugs so far. However, self-assembled peptide nanostructures as carrier have not been used to improve the water solubility of CBD yet. In this study, a self-assembling peptide micelle was demonstrated to be an effective vehicle for encapsulation of CBD and increased its aqueous solubility up to 2000-fold compared to CBD itself.

## 1. Introduction

Cannabidiol (CBD) is a major phytocannabinoid constituent of cannabis. In recent years, CBD has gained attention for its potential therapeutic effects and has been investigated as a drug for various conditions [1]. One of the most well-known uses of CBD is for the treatment of certain types of epilepsy, particularly in children. In fact, a CBD-based drug called Epidiolex was approved by the US Food and Drug Administration (FDA) in 2018 for the treatment of two rare forms of childhood epilepsy, Lennox-Gastaut syndrome and Dravet syndrome. CBD has also been studied for its potential benefits in other conditions, such as anxiety, sleep disorders, pain, and inflammation [2–4]. There have been many clinical and preclinical studies have been conducted to determine the effectiveness and safety of CBD for these uses. In spite of increasing clinical and public interest in using cannabidiol for disease and symptom management, its high lipophilicity and low water solubility limit its effectiveness as a therapeutic [5]. Therefore, it is critical to explore an effective strategy for increasing the water solubility of CBD for its development and application in many fields.

One approach to improve its water solubility and bioavailability is to use carrier systems, which are molecular systems that can chemically and/or physically trap the hydrophobic drug and increase its solubility. Cyclodextrins, nanoemulsions, liposomes, solid lipid dispersions, polymeric systems, and self-assembled peptide nanostructures are well-known carriers that have been used not only to increase the solubility of hydrophobic drugs but also to achieve gradual release of them. It is not surprising that the same or similar delivery systems have been studied to improve the solubility and bioavailability of CBD [6,7]. Among these known strategies, entrapment of CBD into nano and micro-emulsions can be used to create a variety of CBD products, such as tinctures, topicals, and beverages by circumventing its solubility problem.

Nano and micro sized emulsions are lipid-based systems that naturally self-assembles on contact with an aqueous phase into an association colloid carrier system. They can be easily formed by simply mixing drug, lipids and a relatively high concentration of emulsifiers, usually with one or more hydrophilic cosolvents/coemulsifiers [7,8]. Being lipophilic, CBD is entrapped in the oil phase of the emulsions and can disperse in aqueous environment.

Liposomes are another lipid-based colloidal system that can be used as CBD carriers. Both hydrophilic and hydrophobic drugs can reside, either in the internal aqueous core or the lipid bilayer, respectively [9]. Liposomes can be used to improve the solubility and stability of CBD, as well as enhance its absorption and bioavailability. However, liposomes have a reduced ability to localize the active compound in the bilayer, resulting in less efficient encapsulation or active compound loading [10].

Cyclodextrins (CDs) are cyclic oligosaccharides that can be also used to improve the solubility of poorly water-soluble molecules. Therefore, the researchers employed β-cyclodextrin (β-CD) and 2, 6-di-O-methyl-β-cyclodextrin (DM-β-CD) to prepare inclusion complexes(ICs) with CBD to improve the water solubility and dissolution rate of CBD [11–13]. Li et.al reported that the water solubility of CBD in CBD/β-CD IC and CBD/DM-β-CD IC was significantly increased to 0.395 and 14.118 μg/mL, which was enhanced by 17-fold and 614-fold respectively, and the in vitro dissolution rate of CBD was also promoted after complexation [11].

There are also many other studies that used solid lipid nanoparticles (SLNs), polymer micelles, nanoparticles and hybrid nanosystems to encapsulate CBD for delivery purposes [5]. None of them directly reported on certain solubility increment of CBD. However, all studies indicate that an increased CBD release is seen due to increased CBD solubility and improved dissolution properties.

**Figure 1:**
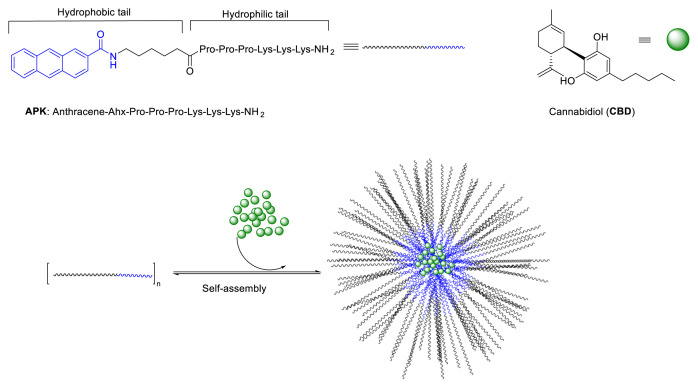
Sequence of APK peptide and structure of CBD molecule. CBD was encapsulated into a micellar peptide carrier via coassembly.

Among above-mentioned carrier systems, self-assembling peptides are playing a more and more important role as a carrier due to their inherent biodegradability, biocompatibility, and ability to self-assembling into various nanostructures [14,15]. Additionally, their high sensitivity to the microenvironment makes them ideal tools for drug delivery [16]. Similar to other examples of aqueous self-assembly, peptide self-assembly is typically built around by an amphiphilic character in monomer units. Amphiphilic peptides having both hydrophilic and hydrophobic domains spontaneously arrange into structures shielding hydrophobic groups by minimizing contact with water. Amphipathicity combined with the functional group richness and modifiability of the peptides enable stable coassembly of some drugs with peptides through strong intermolecular interactions. Due to the aggregation of hydrophobic domains of peptides during self-assembly, these materials may promote drug solubility. Drugs capable of π-π interactions with other aromatic moieties may help to stabilize the assembly [17]. Therefore, successful encapsulation and delivery of some hydrophobic drugs using self-assembled peptide nanostructures have been reported [18–20]. However, so far there is no reported research on using peptide nanostructures for encapsulation CBD. The aim of the current study was to develop peptide-based formulation of CBD that provide higher dispersion than pure CBD in aqueous systems. Considering aromatic content of both CBD and anthracene units, self-assembling APK peptide (Antharecene-Ahx-Pro-Pro-Pro-Lys-Lys-Lys-NH_2_, Ahx: 6-Aminohexanoic acid) has been utilized as a carrier (Figure 1). This newly developed CBD-peptide formulation was characterized with TEM, DLS and FTIR analysis. Increment of solubility of CBD in water was evaluated with HPLC.

## 2. Materials and methods

### 2.1 Materials

CBD (>98%, MW ≈ 314g/mol) was donated by CBDepot.eu (Teplice, Czech Republic). All commercially available Fmoc-protected amino acids, 2-(1H-benzotriazol-1-yl)-1,1,3,3-tetramethyluronium hexafluorophosphate (HBTU), rink amide MBHA resin (0.3–0.6 mmol/g, 100–200 mesh) were purchased from Chem-Impex International (Wood Dale, IL, USA). Piperidine, trifluoro acetic acid (TFA), isopropyl silane (TIS), and solvents (lab-grade and HPLC grade) were obtained from Acros (Beijing, China) and used as received. APK peptides were synthesized, purified and characterized as previously reported (Figure S) [21]. Water was double distilled using a Millipore simplicity 185 (Darmstadt, Germany) purification system (18.1 MΩ) which was fed with distilled water through an internal tank.

### 2.2 Mass spectrometry

High resolution mass spectra (HRMS) of the peptide was recorded on Agilent Technologies 6530 Accurate-Mass Q-TOF. Concentration of the peptide for MS measurement was arranged 0.1 mg/mL with 0.1% formic acid containing ddH_2_O and sample introduction was performed via direct infusion.

### 2.3 High performance liquid chromatography (HPLC)

Preparative reverse phase HPLC purification of peptides was performed as reported earlier [21]. Analytical HPLC of collected fractions were performed on a Dionex UltiMate 3000 HPLC system (Sunnyvale, CA, USA) equipped with a Gemini-NX C18 column- dimensions 150 × 3 mm, particle size 3 μm, pore size 110 Å (Phenomenex, Torrance, CA, USA). Peptides were eluted in acetonitrile containing 0.08% TFA (v/v) and water containing 0.1% TFA (v/v) gradient (5%–100%, 1–35 min., flow 0.4 mL/min). The column oven was kept at 40 °C. After purity of collected fractions confirmed by analytical HPLC, lyophilization was achieved using a Telstar Cryodos Freeze Dryer (Bristol, PA, USA) and peptide was stored at –20 °C.

### 2.4 Fourier-transform infra-red (FTIR)

Using an ATR-FTIR spectrometer (Nicolet iS10, Thermo Scientific, MA, USA), FTIR analysis was performed on commercial CBD and freeze-dried peptide assemblies with/without CBD. A broad scan of the samples was performed from 600 cm^−1^ to 4000 cm^−1^ with a resolution of 0.482 cm^−1^. A total of 32 spectra were averaged to reduce noise. The spectra were recorded in transmittance mode at room temperature. The process of the spectral data was completed with the commercially available software OMNIC Series Software (Thermo Fisher Scientific, MA, USA).

### 2.5 Encapsulation of CBD

APK peptide was dissolved in 100 μL HFIP (1,1,1,3,3,3-Hexafluoro-2-propanol) at varying concentrations below their critical gelation concentrations and over their critical aggregation concentrations. Five μL of CBD solution from 16 mM stock solution was pipetted into each peptide solutions and mixtures were sonicated for 30 min at room temperature. Then, HFIP was left evaporation in an open air for 1h at RT. After the removal of HFIP, peptide amphiphiles were reconstituted in water at pH:4 for 20 min at room temperature. The solutions were centrifuged at 12,000 rpm at 20 °C for 30 min to remove any nonencapsulated CBD. The supernatants were analyzed using analytical HPLC (Dionex UltiMate 3000 HPLC system, USA) equipped with a Gemini-NX C18 column-dimensions 150 × 3 mm, particle size 3 μm, pore size 110 Å (Phenomenex, Torrance, California, USA). Peptides were eluted in acetonitrile containing 0.08% TFA (v/v) and water containing 0.1% TFA (v/v) gradient (5%–100%, 1–30 min., flow 0.4 mL/min). The wavelength detected was 214 nm and the volume of each injection was 10 μL. The concentration of solubilized CBD (mM) in aqueous medium surrounded by a peptide micelle was calculated using a standard CBD curve prepared in advance. Encapsulation efficiency (EE%) of CBD in micelles structure were calculated using the following equations:


$$\text{EE}\%=\frac{\text{Amount of CBD in micelles}}{\text{Total amount of CBD added}}$$

### 2.6 Dynamic light scattering (DLS) measurements

DLS measurements were carried out on a Zetasizer Nano-ZS equipment (Malvern Instruments, USA). Number average hydrodynamic sizes were obtained by cumulative analysis of autocorrelation data. Samples were placed in polystyrene cells, which were cleaned with ultrapure water. Measurements were taken 5 times at 25 °C in order to check their reproducibility. As previously described in the literature, 5 mM peptide solution was prepared in water and pH of the solution was fixed to pH 4 using 1 M HCl solution to obtain peptide micelles [21]. Encapsulation of CBD into micelles were performed using aforementioned solvent coevaporation method.

### 2.7 Transmission electron microscopy (TEM)

Self-assembling peptide micelles impregnated with/without CBD were prepared and then diluted 500 times with water. Three μL of solution was dropped to carbon coated copper grid and then excess sample was removed with a blotting paper after 60 s incubation. Sample coated grid was then wash with 3 μL ddH20. Immediately after removing excess water from grid, 3 μL uranyl acetate (2 wt %) was pipetted onto grid as negative stain and waited 60 s. Excess stain was removed with a blotting paper. Finally, carbon grids were air-dried at RT for at least 3 h before imaging. Light-field TEM imaging was performed on a FEI Tecnai G2 F30 electron microscope operating at an acceleration voltage of 300 kV.

## 3. Results and discussion

### 3.1 CBD encapsulation efficiency of peptide micelles

**Figure 2:**
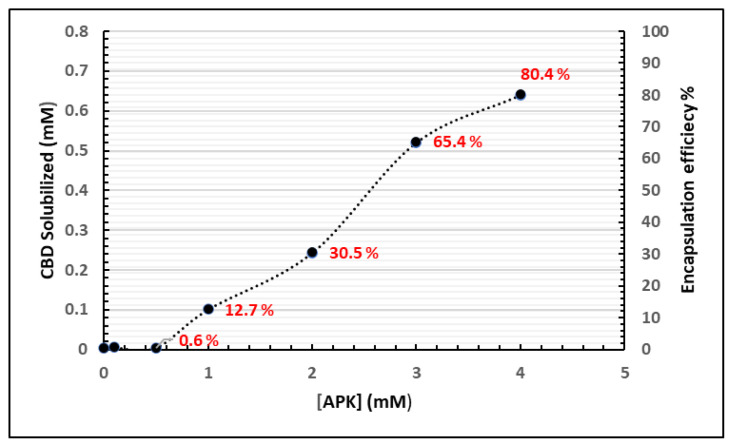
Peptide amphiphile nanomicelle encapsulation of cannabidiol. The initial concentration of CBD was kept constant at 0.795 mM while the concentration of peptide was gradually increased up to 4mM.

To encapsulate CBD into the peptide micelles, a coassembly method was used to yield quantitative levels of encapsulation [19]. In this method, both APK peptide and CBD were mixed in mutually dissolving solvent, which was hexafluoroisopropanol (HFIP). HFIP is a low boiling point solvent and is easily removed by evaporation to produce a film. After the removal of HFIP, the mixture was reconstituted in water. pH of water was adjusted to 4 using few drops of 1 M HCl solution. The amount of CBD loaded was determined by HPLC after removal of nonencapsulated CBD via centrifugation. We found that APK peptide increased a satisfactory CBD solubilization and encapsulation efficiency (Figure 2).

APK peptides are self-assembled into shell-core nanomicelles formed by the hydrophilic (-Lys-Lys-Lys-) and hydrophobic (Anthracene) molecules. Hydrophobic CBD molecules can reside on the core of the shell via π-π stacking and Van der Waals interactions while hydrophilic lysine tails interact with water allowing nanomicelles to solubilize CBD in aqueous medium. CBD shows a low water solubility, measured at 0.1 μg/mL (3.18 × 10^−^^4^ mM) [22,23]. When using the micelle forming APK peptide to encapsulate CBD, aqueous solubility values as high as 0.64 mM CBD were observed, which is more than 2000-fold higher than the solubility reported for CBD alone in water. When encapsulating a 0.795 mM initial concentration of CBD, an efficiency of 80.4% was observed for a 4 mM APK solution. A further increase in peptide concentration did not result in any further increase in the solubility of CBD, but solubility of CBD remained almost same against increased peptide concentration. To the best of our knowledge, 80.4% EE and 2000-fold increase in solubility for CBD are the highest values ever reported using any type of nanocarrier systems (polymer, peptide or inorganic nanoparticles) [24].

### 3.2 Structural analysis of CBD loaded peptide carriers

After encapsulation of CBD into peptides using coassembly method, nonencapsulated CBD was removed with high-speed centrifugation and resulting aqueous mixture was freeze-dried. Beige color solid was analyzed with FT-IR and HPLC to confirm the residence of CBD into the carrier.

**Figure 3:**
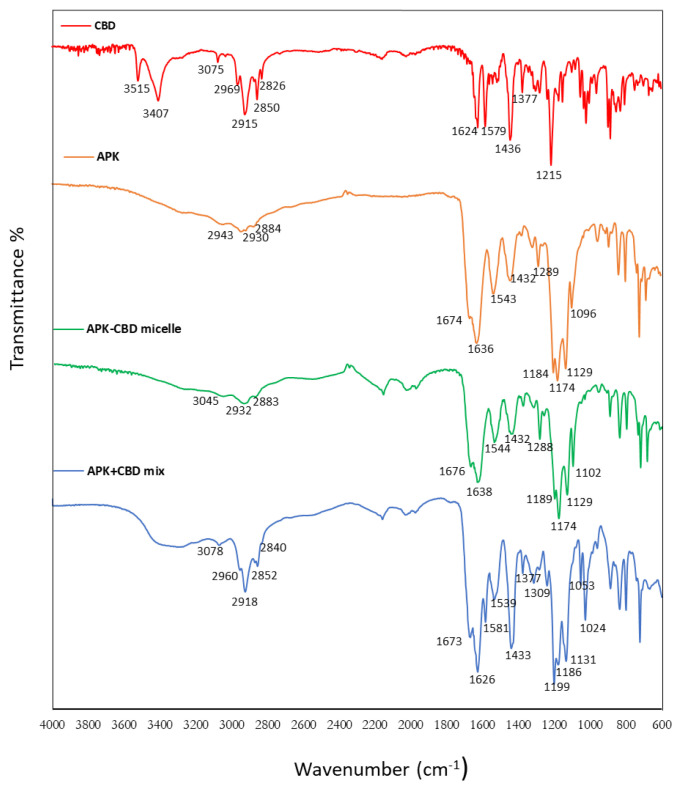
FTIR spectra of the compounds, from top to bottom: CBD, APK peptide, CBD loaded APK micelles and physical mixture of CBD, APK peptide.

FT-IR method was employed for the evaluation of the chemical composition of the micelles after CBD incorporation and the possible interactions between CBD and amino acid side chain of peptides. FTIR spectrum of CBD loaded peptide carrier possessed the characteristic peaks of CBD, confirming the successful loading of CBD. CBD has two distinct peaks observed at 1579 cm^−1^ and 1624 cm^−1^, possibly due to the C=C stretching vibration of the compound and has a characteristic absorption band at 3515 and 3407 cm^−1^, possibly due to the vibration of the phenolic –OH groups [25]. Additionally, peak at 1377 cm^−1^ assigned for the bending vibration of methyl groups, and peak at 1215 cm^−^^1^ denoted for the C-O stretching vibration in CBD molecule [11]. If CBD incorporated into the micelles, some of high-intensity peaks of pure CBD is expected to shift or disappear due to restriction of the above-mentioned groups inside the micelle core. As seen in Figure 3, the characteristic peaks of CBD at 3515 cm^−^^1^, 3407 cm^−^^1^, 1624 cm^−^^1^, 1579 cm^−^^1^, 1377 cm^−^^1^ and 1215 cm^−^^1^ were all disappeared after CBD incorporation into the micelles. It was worth noting that the spectra of APK and APK-CBD micelles were similar to those of APK because of the low content of CBD in the carrier system. Therefore, FTIR spectra of the physical mixture of APK and CBD were also analyzed to show that there is no interaction between CBD and peptide when they were mixed physically. The findings showed that CBD had entirely or partly entered the cavity of the peptide micelles, resulting in that the vibration of the above-mentioned groups was restricted.

**Figure 4:**
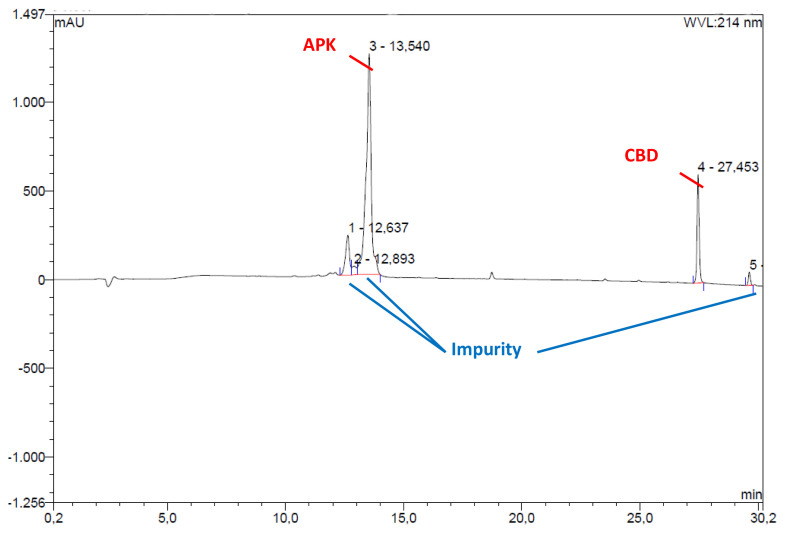
HPLC chromogram of CBD-encapsulated peptide micelles at 214 nm. The APK peptide peak was assigned after the isolation and characterization of peptide with HRMS and CBD peak assignment was based on retention time of commercial CBD standard. HPLC peaks were consigned follows; Peak 1 and 2 are impurities that came along with APK peptide, peak 3 is the APK peptide, peak 4 is the CBD and peak 5 is the decomposition product of CBD.

Encapsulation of the CBD into micelles further endorsed with HPLC analysis. After encapsulation process, carrier peptides that were expected to contain CBD analyzed with HPLC and presence of CBD was confirmed (Figure 4).

### 3.3 DLS analysis of micelles

**Figure 5:**
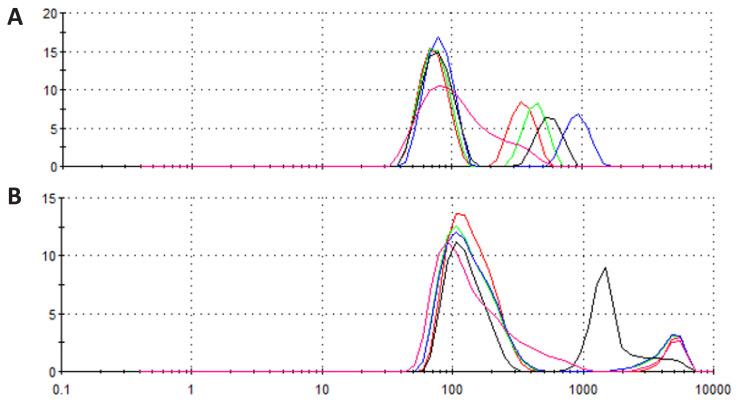
Dynamic light scattering size distribution graph of peptide micelles. (A) Peptide micelles without CBD molecules, (B) CBD encapsulated micelles.

DLS measurements of CBD-loaded micelles were carried out to determine the size distribution of micelles with/without CBD. The intensity-weighted plot revealed a hydrodynamic diameter (Dh) distribution of micelles over a fairly wide range in the range of about 100–1200 nm without CBD molecules (Figure 5A). Average hydrodynamic size of the micelles after CBD incubation were also measured to determine if incorporation of CBD induces swelling or shrinkage of the nanostructure. Similarly, the formation of micelles of different sizes (300–5000 nm) has been observed after CBD encapsulation (Figure 5B). Although not significant, incorporation of hydrophobic CBD into core of micelles which was mainly formed stacked anthracene moieties seems to cause some expansion of the micelle size. Presence of CBD in the core of the micelle seems to cause hindrance for stacking of anthracene molecules and cause some shift of sizes.

### 3.4 TEM analysis of micelles

**Figure 6:**
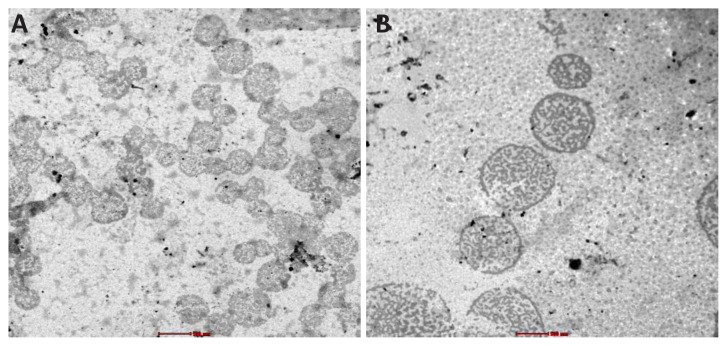
Transmission electron microscopy images. (A) APK micelles, (B) CBD-encapsulated APK using the solvent evaporation method and reconstituted in water. Bar = 500 nm.

After and before encapsulating CBD into the peptide carriers, conventional TEM was used to visualize their individual morphology. TEM images proved that peptides self-assembled to form micellar structure both presence and absence of CBD (Figure 6). Any consistency about size of micelles was not observed. The presence of many large and small micelles was detected in the TEM images. The overall increase in diameter of peptide with CBD compared to peptide alone showed once again, CBD disturbed the stacked anthracene molecules in core. Both DLS and TEM analysis suggested that a small degree of swelling of the micelles might occur in order to accommodate a hydrophobic cargo.

## 4. Conclusion

This work showed that solubility of CBD can be improved up to 2000-fold after inclusion of CBD in self-assembled peptide micelles. CBD was trapped into the peptide micelles using solvent coevaporation method. The presence of CBD in peptide nanostructures were confirmed both in FTIR spectra analysis and HPLC peak assignment. Encapsulation efficiency of peptide micelles and solubility of CBD embedded in micelles were calculated after removal of nonencapsulated CBD from the carrier system. Morphological analysis of peptide micelles with TEM showed that overall micelle size was expanding after the encapsulation. This could be explained as CBD residing in the hydrophobic core is causing sort of disruption of anthracene moieties that forms the interior core.

## Supplementary material

Figure S HPLC chromatogram at 254 nm and electrospray ionization mass spectra of APK peptide. Purity collected peptide fractions was found 89.76% purity. [M+H]^+^ (calculated) = 1010.61913, [M+H]^+^ (observed) =1010.56190.
