# Supplementary figures and images for: Improving the water solubility of cannabidiol using a peptide carrier
Source: Turk J Chem. 2024 Feb 16;48(2):229–36. doi: 10.55730/1300-0527.3655 (PMC11265886; doi:10.55730/1300-0527.3655)

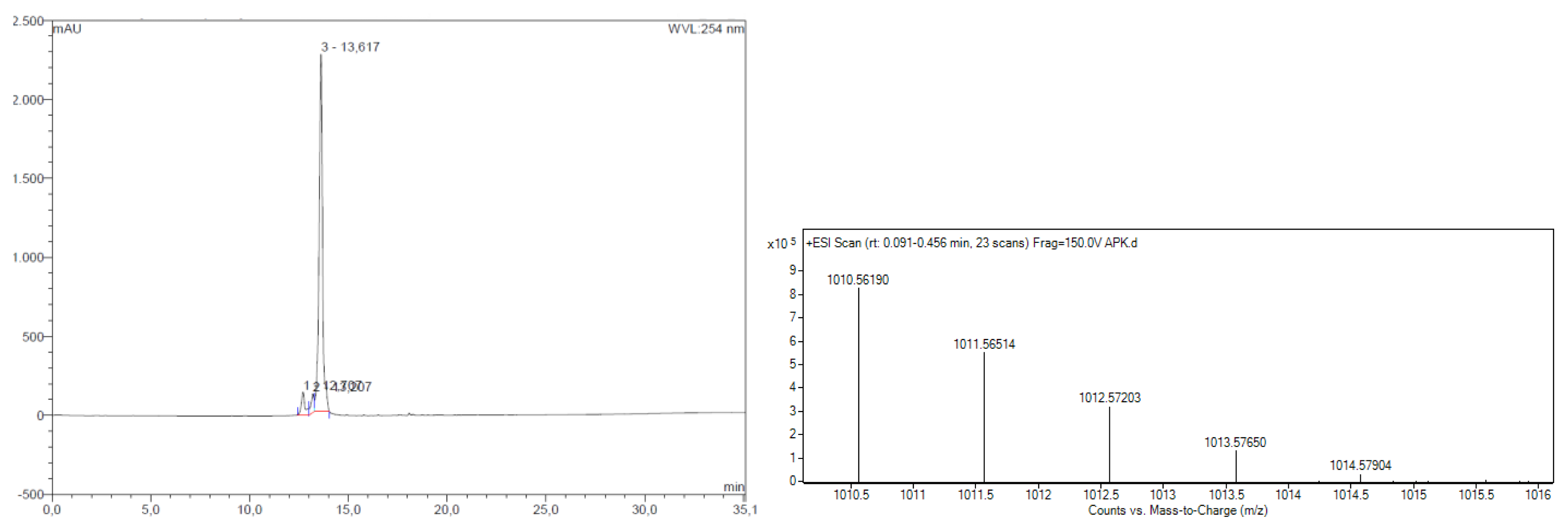

Supplement: Figure S — HPLC chromatogram at 254 nm and electrospray ionization mass spectra of APK peptide. Purity collected peptide fractions was found 89.76% purity. [M+H]+ (calculated) = 1010.61913, [M+H]+ (observed) =1010.56190. [file tjc-48-02-229s1.tif]
